# PEER SUPPORT FOR CAREGIVERS OF AFRICAN AMERICANS LIVING WITH DEMENTIA: A PHASE 1 BEHAVIORAL INTERVENTION PROTOCOL

**DOI:** 10.1093/geroni/igad104.1126

**Published:** 2023-12-21

**Authors:** Karen Moss, Mary Beth Happ, Abraham Brody, Celia Will, Alai Tan, Seuli Bose Brill, Karen Bullock

**Affiliations:** The Ohio State University, Columbus, Ohio, United States; The Ohio State University, Columbus, Ohio, United States; NYU Rory Meyers College of Nursing, New York City, New York, United States; The Ohio State University, Columbus, Ohio, United States; The Ohio State University College of Nursing, Columbus, Ohio, United States; The Ohio State University, Columbus, Ohio, United States; Boston College, Boston, Massachusetts, United States

## Abstract

African American caregivers of older adults living with Alzheimer’s disease or a related dementia are at high risk for physical, spiritual, and psychosocial challenges. Peer mentorship, a relationship-centered person-to-person approach may reduce healthcare decision-making burden among African American caregivers through cultural tailoring by promoting oral traditions, personal contact, and storytelling. The purpose of this Stage 1 intervention study is to use a stakeholder-informed approach in further developing and pilot testing the co-created Peer Support for Caregivers of African Americans Living with Alzheimer’s Disease and Related Dementias (Pair2Care), a culturally sensitive caregiver peer support program. Pair2Care feasibility and acceptability will be evaluated in current (n=15) and trained former (n=15) African American Alzheimer’s and related dementia caregivers. Pair2Care will be deemed feasible and acceptable by evaluating eligibility criteria, recruitment and retention data, protocol adherence, satisfaction, and feedback on program appropriateness for broader dissemination. Preliminary intervention outcomes of caregiver anxiety, depression, quality-of-life, social support, self-efficacy for surrogate decision-making, and intent to use palliative care or hospice services will be assessed to evaluate intervention effectiveness. At 6 months as compared to baseline, current caregivers will report less anxiety and depression, improved quality-of-life, social support, and self-efficacy for surrogate decision-making, as well as intent to use palliative care or hospice services as part of healthcare and advance care planning decision- making. Pair2Care poses an innovative strategy with the potential to address advance care planning and healthcare decision-making, a significant health disparity problem and thereby promote health equity.

